# I Know What to Do; I Can Do It; It Will Work: The Brief Parental Self Efficacy Scale (BPSES) for Parenting Interventions

**DOI:** 10.1007/s10578-023-01583-0

**Published:** 2023-08-25

**Authors:** Matt Woolgar, Sajid Humayun, Stephen Scott, Mark R. Dadds

**Affiliations:** 1 Department of Child and Adolescent Psychiatry, Institute of Psychiatry, Psychology & Neuroscience, King’s College London, London, UK; 2 School of Human Sciences, University of Greenwich, London, UK; 3 School of Psychology, Faculty of Science, University of Sydney, Sydney, NSW Australia

**Keywords:** Parental self-efficacy, Parenting intervention, Disruptive behaviour problems

## Abstract

Parental self-efficacy predicts outcomes for parenting interventions for child behaviour problems, but there is a need for a brief measure that can be repeated over treatment and applies to a wide age range. The present study describes the development of such a measure, the Brief Parental Self-Efficacy Scale (BPSES). The psychometrics of the BPSES is presented across a wide age range from preschool to late adolescent in a sample comprised of four different intervention contexts. Evidence for structural validity, internal consistency, content validity, configural measurement invariance (equivalent factor structure) and test–retest reliability is presented alongside convergent validity against measures of parental self-efficacy, child behaviour problems, as well as self-report and observed parenting styles. Finally, lower levels of BPSES at baseline predicted increased disengagement from an intensive, individualised family therapy intervention for antisocial youth, while higher baseline levels predicted increased response to a group parenting programme for primary school aged children. The BPSES shows promise as a measure that can be used across a wide age-range, for a variety of parenting interventions for disruptive behaviour problems and which is sufficiently brief to be used as a routine outcome measurement during treatment.

## Introduction

Parent training programs are highly effective means to address children’s behavioural problems [29]. Within these programmes carers act as the child’s therapist in the home, implementing the skills learnt in the therapy sessions, and must feel sufficiently competent in their abilities to take on the challenge between sessions, away from the support of therapist or group, and then to maintain effective parenting after treatment stops.

Parental self-efficacy, defined at a general level as a belief in the ability to parent effectively, has been shown to influence a wide range of parenting outcomes including parental well-being, the parent–child relationship and child outcomes [1]. The construct can be considered at a general level, for example confidence in the parenting role overall, e.g., ‘I feel sure of myself as a mother/father’ (e.g., [10]), or as narrow or task-specific self-efficacy, often related to developmentally specific parenting tasks such as breastfeeding or bedtime routines found to be particularly useful for parents with infants or toddlers [6] and children with disabilities who pose specific developmental challenges [17, 23, 38]). In between these levels parental self-efficacy has been defined at a domain-specific level, e.g., relating to discipline [21], and this has been especially useful for thinking about outcomes related to child behaviour problems, especially as children move from toddlerhood to preteens [5]. In contrast, task-specific measures can limit the utility of the constructs over very narrow age-ranges, even within a year, given how rapidly developmental tasks can change [41], while general measures of parental self-efficacy relate more to global outcomes, such as academic success than to the specific challenges of parenting children with disruptive behaviour problems [3].

Parental self-efficacy has been found to be significantly lower in carers referred to parenting clinics compared with community controls [33]. Lower levels of parental self-efficacy are associated with several dimensions of parenting targeted in parenting programs including coercive discipline [4] and reduced sensitivity and warmth [40], as well as with parenting factors that can moderate treatment success, including depression and other features of social disadvantage associated with behavioural problems or parental maltreatment (e.g., [18, 21, 41]).

There are many existing measures for assessing various parental self-efficacy constructs, a recent review identified 34 measures [43], even after discarding 10 from a review conducted a few years earlier [7]. Of these 34 measures, the majority had been designed for specific age-ranges, mostly for infants and toddlers, while only three very broad measures, more about general satisfaction in the parenting role than parental self-efficacy per se, went beyond 12 years of age. Moreover, most of the instruments were relatively long with an average of 26 items. The most widely used measure in parenting programmes [11] is the Parental Sense of Competency Scale (PSOC [20]). However, it is relatively long, comprised of 17 items, and a quality assessment rated the PSOC as slow to administer and with complex sentences (e.g., “I would make a fine model for a new mother/father to follow in order to learn what she would need to know in order to be a good parent”), and in recognition of the scale accessibility issues, some have simplified the standardised version to an 8^th^ grade reading level to better suit diverse and multistressed samples (e.g., [41]).

The current study presents the development of a brief parental self-efficacy scale (Brief Parental Self-Efficacy Scale; BPSES), specific to the domain of effective and supportive disciplining, for the use in parenting intervention studies for child and youth disruptive and antisocial behaviours across a wide age range. The study presents the content validity, structural validity, measurement invariance, internal consistency, test–retest reliability, and several indices of convergent validity of the measure in four different intervention contexts and across a wide age range. Finally, some initial data on its utility for interventions at baseline, including associations with independently observed parental behaviours in the home, as well as treatment engagement and outcomes is reported.

## Method

### Development of the BPSES

A panel of professional experts in parent training comprised of clinical psychologists, child psychiatrists and developmental psychologists reviewed existing literature on parental sense of “confidence, effectiveness, agency” at the domain level in producing positive behaviour change in themselves and their child, distilling the range of ideas appearing in the literature into 3 basic dimensions of Knowledge (“I know what to do”), Ability (“I can do it”), and Outcome (“It will work”). The experts then generated a set of items that represented each of these dimensions. The initial set of items for each domain was reduced by consensus to a final set of 3 items, each comprised of two items with positive valency (e.g., “The things I do make a difference to my child's behaviour”) and one with negative valency (e.g., “Whatever I do my child will remain difficult”), see Table 1. A reading accessibility assessment indicated that the final BPSES items should be easy to read for most adults (Flesch-Kincaid Reading Ease index = 82.9, equivalent to Grade 6th US educational level; [24].

**Table 1:** The brief parental self efficacy scale initial items (final selected items numbered bold)

**1**	Even though I may not always manage it, I know what I need to do with my child (Knowledge)
2	My child's behaviour largely depends on what I do and how I feel (Outcome)
3	Whatever I do my child will remain difficult (Outcome—reversed)
**4**	I am able to do the things that will improve my child's behaviour (Ability)
**5**	I can make an important difference to my child (Ability)
6	I don’t know what I can do to control my child (Knowledge—reversed)
7	I am often too tired or busy to do the things that would make a difference to my child (Ability—reversed)
**8**	In most situations I know what I should do to ensure my child behaves (Knowledge)
**9**	The things I do make a difference to my child's behaviour (Outcome)

In keeping with other domain-specific measures of parental self-efficacy (e.g., [20, 23]) respondents were not asked to recall a specific time period, but asked to say how much they agree or disagree with each one on a five-point Likert scale: strongly disagree; disagree; neutral; agree; strongly agree (corresponding to scores 1 to 5, respectively). Scores were summed within each triad (with the negative valency item reverse coded) creating three subscales ranging from 3 to 15. These 9 items were used in the questionnaires for all 4 studies.

### Samples

Four samples were combined to test the psychometric properties of the new measure. Three assessed parents attending parenting interventions based on social learning theory models, mainly for primary school aged children, while the fourth assessed a family therapy intervention targeted at older children and youth within the forensic system. For the current study, all data for the initial psychometrics’ is pooled (*N*_max_ = 355), unless otherwise stated, to provide information about parental self-efficacy from early childhood through to late teens. Two studies also incorporated observational measures of parent–child interactions pre- and post-treatment which provided independent assessment of parenting style.

The Helping Children Achieve study (HCA; [36]) is a 4-armed randomised controlled study targeting children at risk for antisocial behaviour across two sites in the UK (*N* = 189 at baseline with BPSES scores), comprising a predominantly white sample (157 white British and white other and 31 minority participants: 9 black British, 3 black African, 7 Asian, 12 mixed other and 1 not stated) of 91 girls and 98 boys of mean age 5.86 (SD = 0.53, range 4.42 to 7.14 years). Two of the four arms offered an Incredible Years (IY; [42]) intervention (*n* = 77) and the other two were non-parenting intervention control groups, either signposting only or signposting with a reading intervention (*n* = 112). Assessments were repeated after baseline at two time points for all subjects: within 9–11 months of pre-assessment (*N* = 133; signposting-only controls *n* = 40) and at 22 months (N = 114; signposting-only controls *n* = 37). A subsample of the four arms were also assessed for mediators, including the BPSES measure, at 6 (*N* = 80; signposting-only controls n = 22) and 12 (*N* = 104; signposting-only controls *n* = 23) weeks post assessment.

The Study of Adolescents’ Family Experiences (SAFE; [19]), the UK’s first randomised controlled trial of Functional Family Therapy (FFT,[2]), for youth in contact with the Youth Offending Services comprised 91 families with BPSES data (pre-randomization), of whom the majority were white or white other (one mixed race); and 23 girls and 68 boys, of mean age 14.92 (SD = 1.59, range 10 to 18 years). Post randomization 39 were in a treatment as usual control group and 52 in an FFT intervention group. Questionnaire assessments were repeated for the whole sample, 6 (*N* = 78, 31 in the control group) and 18 (*N* = 76, 29 in the control group) months post assessment.

The Talking & Listening with your Child Study (TLC) is a UK subsample of data reported in Dadds et al. [8] for a feasibility study of a personalised intervention for children with behaviour problems and high levels of callous-unemotional traits. This subsample comprised 18 children (9 white and 9 minority: 3 black and 6 mixed race) 6 girls and 12 boys of mean age 5.84 (SD = 1.19, range 4.1 to 7.8 years) and baseline data is presented only.

The Community Parenting Group study (CPG) consisted of parents of primary and secondary school aged children referred to Incredible Years parenting groups run in community centres in South London by voluntary agencies (*N* = 57). The sample comprised (33 white British or white other and 23 minority: 12 black British or black African, 4 Asian, 7 mixed race and 1 did not identify) of 40 girls and 17 boys of mean age 7.78 (SD = 3.05, range 2.1 to 15.1 years). Questionnaire data was collected pre and post intervention as part of a doctoral thesis [28], but only baseline data is presented here.

There is a wide age range in these samples, and the majority of the older children were in the SAFE sample, a functional family therapy intervention (FFT) for forensic youth (*N* = 91, mean age = 14.92, SD = 1.59, range 10 to 18), whereas the rest of the predominantly younger samples (HCA, TLC and CPG, *N* = 264) were from social learning theory (SLT) intervention samples (mean age = 6.28, SD = 1.68, range 2 to 15). Indeed, the forensic sample differed on all demographics measures and questionnaire outcomes compared to the SLT sample being higher risk and having more negative outcomes (all p < 0.01), except for corporal punishment which was higher in the younger age group (p < 0.001). The following analyses are presented for the whole sample, and also split according to this distinction between SLT and FFT which captures differences in intervention type, severity of behaviour problems (i.e., forensic vs non-forensic) as well as substantial age differences.

### Measures

Strengths and Difficulties Questionnaire [16], a widely used tool with established reliability and validity, measures the adjustment and psychopathology of children and adolescents. Parent report data from the conduct problems and prosocial behaviour subscales is presented here, completed for all four studies (*N* = 350).

Short Alabama Parenting Questionnaire (APQ; [34, 35]), 15 items measure five subscales relevant to child conduct problems, two positive scales (parental involvement and positive parenting) and three negative scales (poor supervision, inconsistent discipline and corporal punishment) completed in the HCA, TLC and SAFE studies (*N* = 296).

Depression and Anxiety Stress Scale (DASS; [27]) comprises 21 items measuring depression, anxiety and stress. Here we present only the depressed mood scale, completed in the HCA, TLC and SAFE studies (*N* = 293).

Parental Sense of Competence (PSOC; [20]) is a 17-item measure of parental self-efficacy, producing an overall parental sense of competence score, as well as efficacy and satisfaction subscales, completed in the CPG study only (*N* = 56).

The Antisocial Process Screening Device (APSD; [13]) measures three traits found to be important for antisocial youth (Callous-unemotional traits, Narcissism and Impulsivity), only the Callous-unemotional traits subscale is reported here, completed in the HCA, TLC and SAFE studies (*N* = 295).

### Observations of Parent–Child Interactions

In the HCA study a 15-minute direct observation of parent–child interaction across three tasks (child-directed free play; parent-directed building task using Lego blocks; a tidy-up task) was available for *N* = 176 families at baseline who had BPSES data, and for *N* = 115 families who also had follow up data at 22 months. Each episode was videotaped and later coded by raters blind to identifying information using (a) Coding of Attachment-Related Parenting (CARP; [30]), a measure of maternal interaction quality on 3 dimensions relevant to attachment quality: sensitivity; positivity; and negativity, and (b) Parent Behavior Coding Scheme (PBCS; [34, 35]) an event-based observational measure adapted from the widely-used Behavior Coding Scheme [12] for measuring the mechanisms of change in SLT programmes, on four types of events: effective (alpha) commands; ineffective (beta) commands; positive attends/praise; and criticisms.

In the SAFE study, a structured interaction using a standard 10-minute hot topic problem-solving paradigm was recorded in which the parent and adolescent discuss a topic chosen by each of them that is a leading source of conflict in the relationship and later coded by independent raters [37]. Positive and negative interactions, for each of parent and youth, were coded on a 5-point Likert scale that best reflected the participant’s overall behaviour in each interaction task, creating dimensions of positivity and negativity. Observations were available for 69 families at baseline who had BPSES data.

### Statistical Analyses

All data analyses were conducted with IBM SPSS V 28.0 and IBM SPSS Amos V 27.0 statistical software. Structural validity was explored using Confirmatory Factor Analysis (CFA) to test the model fit of the 3-factor solution for the whole sample. Thereafter measurement invariance across two subgroups (SLT and FFT) was assessed in the CFA first by the quality of fit of an unconstrained model (configural invariance) and then for metric invariance whether a constrained measurement model did not differ from the unconstrained model [31]. Internal consistency was represented by Cronbach’s alpha on the whole sample. Test-rest reliability was explored within each of the two RCT samples (HCA and FFT) presenting the correlations between baseline scores in the control groups with follow up scores at 6 and 12 weeks and also 10 and 22 months in the younger sample, and 6 and 18 months in the older sample. Convergent validity was assessed by the correlation between the BPSES score and the PSOC, an established and widely used measure of parental self-efficacy, within the CPG sample at baseline.

Application of the BPSES in the context of two RCTs was conducted within the HCA and FFT samples. First, associations between BPSES with engagement and observations in the home were explored using correlational analyses. Secondly, within the HCA study only, the impact of treatment on parental self-efficacy was assessed using a mixed model ANOVA comparing treatment and control group outcomes from baseline to (a) post-treatment and (b) follow up. Finally, within the HCA study only, correlations between baseline BPSES with simple pre and post changes scores on (a) behaviour problems and (b) observations of parenting behaviour were calculated.

For the initial CFA analyses of the nine BPSES items there was 0.04% missing data which were determined to be missing completely at random using Little’s MCAR test (p = 0.17). The item-level data were then extrapolated using the EM algorithm for missing value analysis in SPSS before importing to AMOS. For all other analyses, where data is missing pairwise comparisons are reported.

All analyses using the full sample of four pooled subsamples (*N* >  = 350) were powered to detect to detect small to medium effect sizes (e.g., r > 0.20 and d > 0.30) at beta = 0.8, and also for comparisons between the SLT and FFT subsamples. However, for the further subsample analyses medium effects sizes were indicated for a similar level of power (e.g., within the FFT sample for r > 0.33, for beta = 0.8).

## Results

### Structural Validity

An initial Confirmatory Factor Analysis (CFA) indicated the a priori 3 factor model based on Knowledge, Ability and Outcome was a poor fit (CMIN/DF = 7.22; CFI = 0.793; RMSEA 0.132) to the data, and the 3 factors showed a high degree of inter-correlation (0.79 to 0.90) suggesting a single factor solution would fit the data better. A model with all 9 items was also a poor fit (CMIN/DF = 6.90; CFI = 0.779; RMSEA 0.128) and inspection of standardised regression weights, as well as error variances indicated problems with the 3 reversed scored items (items 3, 6 and 7), and also item 2, suggesting a single factor structure of five items 1, 4, 5, 8 and 9.

This 5-item model was a reasonable fit to the data (CMIN/DF 4.20; CFI 0.960; RMSEA 0.094). To check for stability across the wide age range, the model was rerun allowing for different models according to the SLT and FFT groups. The grouped model was a good fit to the data (CMIN/DF 2.62; CFI 0.957; RMSEA 0.068) thereby demonstrating configural invariance across the two groups. Next, metric invariance was tested by constraining the factor weights to be equal, but this model differed significantly from the unconstrained model (CMIN = 10.32, df = 4, p = 0.03) indicating that the BPSES did not show metric invariance across the two groups that differed by both age and problem severity.

### Internal Consistency

The resulting single scale of five items had satisfactory internal consistency (Cronbach’s alpha = 0.75), with a possible range of 5 to 25, and was normally distributed in this sample (*N* = 355, mean 19.30, SD = 3.29, range 5 to 25).

### Test–Retest Reliability

Test–retest reliability was assessed in the two RCT studies that had pre and post treatment, as well as follow up data, for the control groups. In the younger HCA sample the control group (signposting only, not the literacy group) was tested at 6 weeks (r_28_ = 0.73), 12 weeks (r_23_ = 0.78) as part of the mediators sample, and at 10 months (r_40_ = 0.55) and 22 months (r_19_ = 0.41) post initial assessment, suggesting high levels of stability in the absence of any intervention, especially over the short term, i.e., the duration of a 12-week intervention. Within the SAFE study the control group was tested at 6 (*N* = 22) and 18 (*N* = 23) months post-initial assessment, and the stability was also high (r_22_ = 0.66 and r_23_ = 0.78, respectively).

### Convergent Validity

The association between the BPSES and a well-established measure of parental self-efficacy (PSOC), was assessed in the CPG sample and revealed a scale convergence with the overall PSOC score (r_56_ = 0.46, p < 0.001).

For the sample as a whole, greater parental self-efficacy was associated with parent reports on the SDQ of lower child conduct problems (r_350_ = - 0.32) and higher prosocial skills (r_349_ = 0.28) for both age groups (Table 2). Lower parental self-efficacy was associated with higher CU traits in the older FFT sample (r_91_ = - 0.35) but not for the younger SLT sample (r_204_ = - 0.06), indeed mean CU traits were significantly higher in the forensic FFT sample (5.92, SD = 2.31 vs 4.93, SD = 3.34, t_293_ = 2.56, p < 0.05, d = 0.32).

**Table 2:** Pearson correlations between BPSES scores and questionnaire covariates, for the whole sample, and within the social learning theory and functional family therapy programme subsamples

	All	*N*	SLT	*n*	FFT	*n*
DASS						
Carer mood	− 0.22**	293	− 0.15*	202	− 0.14	91
APQ						
Parental Involvement	0.45**	293	0.22**	203	0.47**	90
Positive Parenting	0.20**	296	0.05	205	0.23*	91
Poor Supervision	− 0.16*	295	− 0.09	204	− 0.13	91
Inconsistent Discipline	− 0.04	296	− 0.18**	205	− 0.13	91
Corporal Punishment	− 0.15*	294	−0.25**	203	− 0.11	91
SDQ						
Conduct problems	− 0.32**	350	−0.23**	259	− 0.27**	91
Prosocial skills	0.28**	349	0.23**	258	0.34**	91
APSD						
Callous Unemotional Score	− 0.17*	295	−0.06	204	− 0.35**	91

*SLT* social learning theory based group, *FFT* functional family therapy group, *DASS* depression and anxiety stress scale, *APQ* alabama parenting questionnaire; *SDQ* strengths and difficulties questionnaire; *APSD* antisocial process screening device

**p < .01; *p < .05

Parenting style assessed by the APQ subscales revealed notable associations between parental self-efficacy and parental involvement which was associated with higher self-efficacy, especially for the FFT sample (r_90_ = 0.47 vs r_203_ = 0.22, respectively). Similarly, positive parenting was associated with higher parental self-efficacy in the FFT sample only. By contrast, corporal punishment was negatively associated with parental reports of efficacy but only for the younger SLT group (r_203_ = - 0.25 vs r_91_ = − 0.11, respectively). The three measures that showed differing convergent validity between the two samples, maternal involvement, positive parenting and corporal punishment, all had significantly higher mean scores in the younger HCA sample (12.72, SD = 1.62 vs 7.45, SD = 2.12, t_291_ = 23.21, p < 0.001, d = 2.93; 12.45, SD = 3.49 vs 9.79, SD = 2.11, t_294_ = 6.73, p < 0.001, d = 0.84; and 4.54, SD = 1.59 vs 0.50, SD = 0.97, t_292_ = 22.29, p < 0.001, d = 2.81, respectively). There were no notable associations (e.g., r ≤ 0.20) between the other dimensions of parenting measured by the APQ.

### Associations with Demographic, Parent and Child Variables

For the whole sample, parents of older children had lower parental self-efficacy (r_353_ = − 0.26 p < 0.01), but parental self-efficacy was not associated with age within the FFT or SLT samples (r_91_ = − 0.05 and r_262_ = − 0.07, respectively), and indeed the older, FFT sample showed significantly lower levels of self-efficacy at baseline (17.83, SD = 3.29 vs 19.80, SD = 3.15; t_353_ = 5.08, p < 0.001, d = 3.18).

Table 3 presents the mean levels of parental self-efficacy according to the remaining demographic data. Parental self-efficacy was not associated with child sex or ethnicity, nor with being a lone carer. However, parents with lower educational attainment reported lower parental self-efficacy, but this was only the case within the younger, SLT sample (18.73, SD = 3.25 vs 20.16, SD = 3.05, t_211_ = − 3.15, p < 0.01). Finally, maternal mood had a modest impact upon parental self-efficacy scores (Table 2, r293 = 0.22) overall, but this effect was small in each of the age groups.

**Table 3:** BPSES scores by sample characteristics for whole sample, and within the social learning theory and functional family therapy programme subsamples

	All (355–303)				SLT (264–213)				FFT (91–90)			
	Mean	SD	*N*	t-value	Mean	SD	*n*	t-value	Mean	SD	*n*	t-value
Sex												
Female	19.54	2.95	160	t_353_ = 1.25	19.88	2.83	137	t_262_ = 0.40	17.52	2.88	23	t_89_ = − 0_._52
Male	19.10	3.55	195		19.72	3.47	127		17.94	3.43	68	
Ethnic Minority												
White	19.24	3.14	287	t_352_ = 69	19.89	2.84	197	t_261_ = 0.77	17.81	3.30	90	t_89_ = − 0.65
Minority	19.55	3.92	67		19.54	3.95	66		20.00	–	1	
Lone carer												
No	19.50	2.96	194	t_257.1_ = 1.67	19.90	2.95	152	t_242_ = − 0.86	18.04	2.53	42	t_89_ = − 0.56
Yes	18.88	3.77	141		19.54	3.59	92		17.65	3.84	49	
Education > 16												
No	18.33	3.35	131	t_301_ = − 3.70**	18.73	3.25	73	t_211_ = − 3.15**	17.82	3.42	58	t_88_ = −0.06
Yes	19.73	3.19	172		20.16	3.05	140		17.87	3.14	32	

*SLT* social learning theory based group, *FFT* functional family therapy group

**p < .01

### Application to Intervention Contexts

Two of the studies were conducted under RCT conditions which permitted additional analyses: (a) engagement and/or drop out from the interventions; (b) observational assessments of parent–child interactions at baseline in relation to their concurrent parental self-efficacy; (c) change in parental self-efficacy during treatment; and (d) whether baseline parental self-efficacy scores predicted response to treatment.

#### Engagement

For the younger age group (HCA sample) parental self-efficacy at baseline did not predict the number of sessions attended (r_60_ = − 0.09, ns), nor were there differences in baseline parental self-efficacy for those who did (*n* = 52) versus did not (*n* = 8) attend at least half of the 12 sessions (20.48, SD = 2.17 vs 20.62, SD = 3.24, t_58_ = 0.16, ns).

In the FFT sample, for the 52 cases with baseline parental self-efficacy scores who entered treatment there was no association between parental self-efficacy and the total number of sessions attended in the treatment group (r_52_ = 0.15, ns). However, the FFT programme has engagement and motivation sessions prior to the behaviour change sessions, and those who failed to progress to the behaviour change phases from the engagement sessions had significantly lower self-efficacy scores at baseline (15.83, SD = 2.44 vs 18.42, SD = 3.28, t_50_ = 2.52, p < 0.05, d = - 0.83).

#### Observations

For the younger, HCA sample, the event-based coding scheme revealed higher parental self-efficacy was associated with more effective instructions (alpha commands) at baseline (r_173_ = 0.18, p < 0.05) but not with ineffective instructions (beta commands, r_175_ = 0.03) nor with other event-based indices of interactions such as attending/praise (r_175_ = 0.05) or criticisms (r_176_ = - 0.03). Parental self-efficacy was not associated with any baseline global ratings of parental sensitivity (r_174_ = − 0.04), positivity (r_175_ = 0.00) or negativity (r_175_ = 0.03).

For the older, FFT sample, higher parental self-efficacy was associated with marginally lower observed baseline parental negative behaviour (r_79_ = − 0.19, p < 0.10), but not with positive parenting (r_79_ = - 0.02), nor with positive (r_79_ = − 0.09) nor negative (r_79_ = - 0.03) youth behaviours.

#### Change in Parental Self-Efficacy with Treatment

The treatment used in the HCA study, had a positive impact on child behaviour problems, sustained up to 2 years later [36]. The two treatment groups (n = 50) showed a marginally greater increase in parental self-efficacy (F(1,129) = 3.64, p < 0.10, eta = 0.027, observed power 0.47) immediately post-treatment than the control group (signposting or literacy *n* = 81), but this small effect of treatment did not sustain to follow up at 22 months (F(1,109) = 0.01, ns; control, *n* = 69 vs any IY, *n* = 42).

No treatment effect for youth behaviour problems were found in the first trial of FFT in the UK [19], and similarly no differences in self-efficacy scores were found for the intervention and control group pre and post intervention (F(1,76) = 0.44, ns, observed power 0.10).

#### Change in Child Behaviour Outcomes by Baseline Parental Self-Efficacy

A further hypothesis was that baseline parental self-efficacy may enable parents to maximise their response to treatment; given the lack of treatment effect in the FFT trial, only the HCA trial is considered here. Simple outcome change scores were calculated by subtracting baseline scores from post-treatment scores. Table 4 shows higher baseline parental self-efficacy was associated with parent-reported reductions in child behaviour problems (r_42_ = − 0.34, p < 0.05) and increase in pro-social behaviours (r_42_ = 0.39, p < 0.01), but only for the treatment group. Similarly, within the treatment group only, higher baseline self-efficacy was associated with an increase in parental positive interactions (r_39_ = 0.32, p < 0.05), as well as marginal increases in sensitivity (r_39_ = 0.27, p < 0.10) and the number of attends and praise events at follow up (r_39_ = 0.27, p < 0.10). Those with higher baseline self-efficacy were especially able to make use of a standard parenting intervention to improve both child behaviours and observed parenting quality.

**Table 4:** Correlations of baseline BPSES scores in the HCA sample with changes in parental reports of child behaviour (SDQ) and observed parental behaviour (CARP & PCBS) within treated and untreated groups

	SDQ			CARP				PCBS				
	Conduct	Prosocial	*n*	Sensitivity	Positive	Negative	*n*	Alpha commands	Beta commands	Attends & praise	Criticisms	*n*
Control	− 0.01	0.03	70	0.16^a^	− 0.06	− 0.12	66	0.02	− 0.15	− 0.10	0.07	64
Treatment	− 0.34*	0.39**	42	0.27^+^	0.32*	− 0.10	39	0.09	0.21	0.27^+b^	− 0.05^b^	39

*SDQ* strengths & difficulties questionnaire, *CARP* coding of attachment-related parenting, *PCBS* parent behaviour coding scheme

**p < 0.01, *p < 0.05, + p < 0.10

^a^N = 65

^b^N = 40

## Discussion

This paper reports the development and psychometric properties of a new brief measure of parental self-efficacy (BPSES), demonstrating its applicability across a wide age range and intervention contexts. The final five-item scale had good content validity and sufficient structural validity, as well as acceptable internal consistency. Convergent validity was demonstrated with an established but longer measure of parental self-efficacy, and further supported by associations with child behaviour characteristics (e.g., problem severity) as well as self-report and observed parenting measures. Test–retest reliability was shown for durations up to two years apart, and for the younger SLT sample in particular, the test–retest scores were especially high over the briefer 6-week and 12-week intervals, within the typical durations of parenting interventions for this group. Many of these effects were found across both the younger and older sub-groups, in which the latter also presented with greater problem severity, but as a caveat, while there was evidence of configural invariance across these two distinct groups, metric invariance was not demonstrated.

The domain-specific level of parental self-efficacy used here avoids the developmental restrictions of task-specific parental self-efficacy measures [41] and avoids the broader general level of self-efficacy which tends to extend beyond parenting tasks to greater associations with other aspects of parental personality. Although the factor structure was the same across a wide age-range, parents of older children, with more severe problems reported lower levels of self-efficacy, reflected in the lack of metric invariance (i.e., that each item contributes to the overall construct to the same extent). Indeed, parental self-efficacy tends to reduce from late childhood onwards, although it continues to predict parenting practices [15], and the BPSES did continue to show convergent validity for the older forensic sample. Metric noninvariance can have a substantive meaning, for example, it is unlikely that two such dissimilar groups would form part of the same intervention study. While the BPSES was found to have the same factor structure across the two groups we cannot be confident that self-efficacy in parents of older children in the forensic system means the same as self-efficacy in parents of younger children with less severe problems. Indeed, the parents of the younger children reported substantive differences in their parenting practices, with substantially more involvement, positivity and corporal punishment than the parents of the older children.

The associations with demographics were considered for the sample as a whole, but also within the two subsamples defined by younger children receiving social learning theory approaches, and older children in contact with the forensic system. Parental self-efficacy did not systematically vary by sex, ethnicity or family structure; but for the younger sample only, parents with education beyond 16 had higher levels of parental self-efficacy. There was also a small effect with lower self-efficacy associated with lower mood, but within each age subsample these were small effects. Overall, this measure of parental self-efficacy showed less demographic variation than many other measures [11].

Consistent with previous research (e.g., [1], parental self-efficacy was associated with parental reports of higher conduct problems and fewer prosocial skills across both subsamples. There was a notable association between lower levels of parental self-efficacy and higher child callous unemotional traits, but only in the older forensic sample. A further age-specific effect found previously [9] was that higher levels of parental self-efficacy were associated with lower levels of corporal punishment, but only in the younger age group.

There was some validation of the self-report measure corresponding to difficulties in the parenting relationship observed at home in ways relevant to the sense of being in control of the parenting situation, again varying by age group. For younger children, lower parental self-efficacy was associated with fewer effective (alpha) commands; giving clear and effective instructions to small children is highly effective [32] but does require confidence that these instructions will be followed through. However, there was no association between baseline reports of parental self-efficacy, and more general attachment-related dimensions of positive parent–child interaction at baseline, nor of the SLT indices of positive attends and praise. To some extent this is surprising, given a key task of parenting programmes at this age is to build up positive interactions, but they are typically not a primary treatment component in their own right [25]. For the older sample, lower parental self-efficacy was marginally associated with higher rates of negative parental interactions with their child in a problem-solving discussion, but not with positive parental characteristics, nor with the quality of the youth’s contribution to the task. Indeed, parental negativity and rejection are associated with youth delinquency more than involvement or warmth [26].

The clinical utility of the BPSES was further explored in the two RCT samples. Parental self-efficacy scores at baseline were significantly lower for those parents who dropped out from active treatment phases following the engagement phase of FFT, suggesting that this brief measure could be used to identify those at elevated risk of disengagement and increased efforts targeted at engagement to these families early on. There was no clear effect on engagement with baseline parental self-efficacy in the younger cohort, other than a non-significant trend of higher baseline self-efficacy associated with more sessions attended. Future studies could explore the relationship between parental self-efficacy and engagement in larger samples, with higher rates of disengagement.

For the parents receiving an IY intervention, parental self-efficacy increased slightly over the course of treatment, compared with the control groups, but this small effect did not sustain to follow up. No effects were seen in the older sample, but this RCT did not show effects for the FFT intervention overall, and any possible effect may have been attenuated by the trend of increased drop out with lower self-efficacy scores.

Finally, some simple change scores were calculated for outcome measures for the parents receiving an IY intervention. Within the treatment group only, those with higher baseline parental self-efficacy showed greater improvements with treatment for both parental reports of child behaviour problems and also with observed increases in parental positive interactions and marginal increases in both parental sensitivity and the numbers of praise and attends, indicating that lower baseline parental self-efficacy scores, predicted reduced treatment response.

The current study derived the initial nine items from an expert panel with knowledge of the extensive self-efficacy literature but did not involve parents as part of the process of distilling down a large number of items from long measures to a smaller concise set, and this will have had an impact upon the relevance, comprehensive and comprehensibility domains of content validity which prioritises patient views (cf. COSMIN guidance, e.g., [14]. Thus while the final version of the BPSES is very brief and demonstrated good readability scores promoting comprehensibility, the content validity should be further tested in future studies with parents.

While there was a wide age range and treatment context, the intervention for the older sample was not effective and therefore it was not possible to relate baseline parental self-efficacy to treatment outcomes. Furthermore, the forensic sample was also overwhelmingly comprised of white majority participants, and although previous research has indicated that ethnicity is not a reliable influence on parental self-efficacy [11], applications to more diverse samples, and especially in relation to treatment outcomes, is indicated (and would permit further psychometric testing of measurement invariance).

Although the study was adequately powered for the full sample, and for the comparisons between the SLT and FFT groups, the further testing in the intervention contexts lacked power at conventional levels and must be considered exploratory.

Finally, the current study investigated parental self-efficacy in the context of four intervention studies, but not within prevention settings, within which many parenting interventions take place. Further research will be needed to explore the utility of this measure in indicated samples and for universal parenting programmes.

## Summary

The BPSES provides a brief assessment of domain-specific parental self-efficacy in disciplining interactions, that demonstrates an encouraging range of psychometric properties for use across a wide age range and different intervention contexts, with high stability over durations typical of parenting interventions, and thus suitable as a routine outcome measure during the course of treatment. Baseline scores can be useful to identify those who are more likely to engage in treatment, especially in older samples, but also that those with lower initial levels, may be less likely to respond to evidence-based treatments.

## Data Availability

Not applicable.
